# Infectious Multisegmental Spondylodiscitis in a Swine Farmer: Diagnostic and Therapeutic Insights

**DOI:** 10.7759/cureus.75923

**Published:** 2024-12-18

**Authors:** Joana Castro Vieira, Mafalda Maria Santos, João Vieira Afonso, Mariana Simão de Magalhães, Ana Cristina Teotónio

**Affiliations:** 1 Internal Medicine, Unidade Local de Saúde do Oeste – Hospital Distrital de Caldas da Rainha, Caldas da Rainha, PRT

**Keywords:** infectious spondylodiscitis, magnetic resonance imaging, occupational disease, spinal diseases, spondylodiscitis, streptococcus suis

## Abstract

Infectious spondylodiscitis is an infection of the spine that carries considerable clinical and socioeconomic consequences. Its diagnosis and treatment are complex, and the condition is potentially debilitating. Early diagnosis, including microbiological and, ideally, histological assessment of the affected tissue, is essential to quickly initiate the most appropriate therapy. We present the case of a 57-year-old patient, a swine farmer, with multisegmental spondylodiscitis caused by *Streptococcus suis*. His initial presentation included fever and low back pain. The aim of this clinical case is to emphasize that early diagnosis and treatment are crucial in reducing the rate of permanent neurological deficits.

## Introduction

Infectious spondylodiscitis is an infection of the spine that encompasses vertebral osteomyelitis, spondylitis, and discitis. The incidence of spondylodiscitis varies from 0.2 to 2.4/100,000 per annum, increasing with age and predominantly affecting male patients over 50 years old [1]. It is a condition with major clinical and socioeconomic impact, and its diagnosis and treatment are challenging [2,3].

Despite advances in therapeutic and diagnostic techniques, spondylodiscitis remains associated with high morbidity and mortality rates, remaining a clinically challenging entity. The most common symptom is low back pain, often without specific associated signs. Low clinical suspicion combined with the limited sensitivity of conventional imaging techniques, such as radiography, often results in a delayed diagnosis [4]. Computed tomography (CT) has higher sensitivity than radiography for bone destruction and abscesses, although it can also be normal in the early stages. Magnetic resonance imaging (MRI) of the spine is the gold standard, with a sensitivity of 90%-96%, allowing diagnosis in the early stages and identification of possible complications [5].

Microbiology and histopathology have the potential to establish the etiological diagnosis and guide treatment; however, there are some limitations, such as the time required for pathogen isolation and the difficulty in obtaining tissue for histopathological confirmation [6].

Disease progression can lead to physical deformities and neurological deficits. The main goals of clinical management include pain relief, antibiotic therapy, spinal immobilization, and, when indicated, surgical exploration for biopsy, neural decompression, and/or stabilization of the spine [7].

The aim of this clinical case is to emphasize the need for early recognition of infectious spondylodiscitis, particularly in patients with occupational risk factors, and highlights the critical role of microbiological and histological assessments in guiding prompt targeted therapy. Timely intervention is essential to minimize permanent neurological deficits and improve clinical outcomes.

## Case presentation

We present the case of a 57-year-old male patient, a swine farmer, with a medical history of hypertension, managed with candesartan 16 mg plus hydrochlorothiazide 12.5 mg once daily, and a smoking history of 40 pack-years.

The patient was admitted to the emergency department with intense low back pain, rated 7/10 on the pain scale, aggravated by standing and unrelieved by analgesics, associated with fever lasting for five days. During the physical examination, the patient remained alert, orientated, and cooperative, with normal blood pressure, heart rate, and respiratory rate for his age, with a tympanic temperature of 38.2°C. The neurological examination revealed no motor or sensory deficits.

Laboratory tests revealed leukocytosis (11,000/uL with a predominance of neutrophils), an erythrocyte sedimentation rate (ESR) of 120 mm, C-reactive protein (CRP) of 10.3 mg/dL, and procalcitonin of 1.13 mg/dL (Table 1). The etiological workup, including interferon-gamma release assay (IGRA), angiotensin-converting enzyme (ACE), *Brucella abortus* IgG and IgM, *Coxiella,* and *Borrelia burgdorferi*, was negative.

**Table 1:** Laboratory investigations CBC, complete blood count; WBC, white blood cells; LFT, liver function test; AST, aspartate transaminase; ALT, alanine transaminase; KFT, kidney function test; BUN, blood urea nitrogen; Na, sodium; K, potassium; LDH, lactase dehydrogenase; CRP, C-reactive protein; ESR, erythrocyte sedimentation

Test	Observed Value	Reference Range
CBC		
Hemoglobin	13.4 g/dL	13.6-18.0
WBC	11.00 x 10³/uL	4.0-10.0
Neutrophils	8.8 x 10³/uL	1.5-8.0
Platelets	89 x 10³/uL	140.0-440.0
LFT		
Total bilirubin	0.75 mg/dL	0.20-1.20
AST	29	5-34
ALT	22	0-55
KFT		
BUN	31 mg/dL	18-55
Serum creatinine	0.45 mg/dL	0.70-1.30
Na	130 mEq/L	136-145
K	3.1 mEq/L	3.5-5.1
Additional Tests		
LDH	232	125-220
CRP	10.3 mg/dL	< 0.5
Procalcitonin	1.13 ng/mL	<0.5
ESR	120 mm	12-14

**Figure 1:**
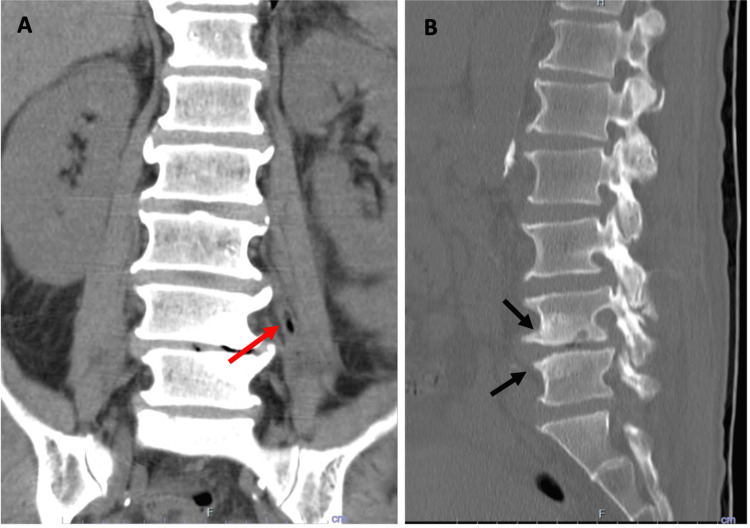
Non-contrast lumbar CT A - coronal view showing the presence of intramuscular air, suggestive of an infectious process (red arrow); B - sagittal view, densification of the bone structure surface (black arrows). CT - Computed tomography

**Figure 2:**
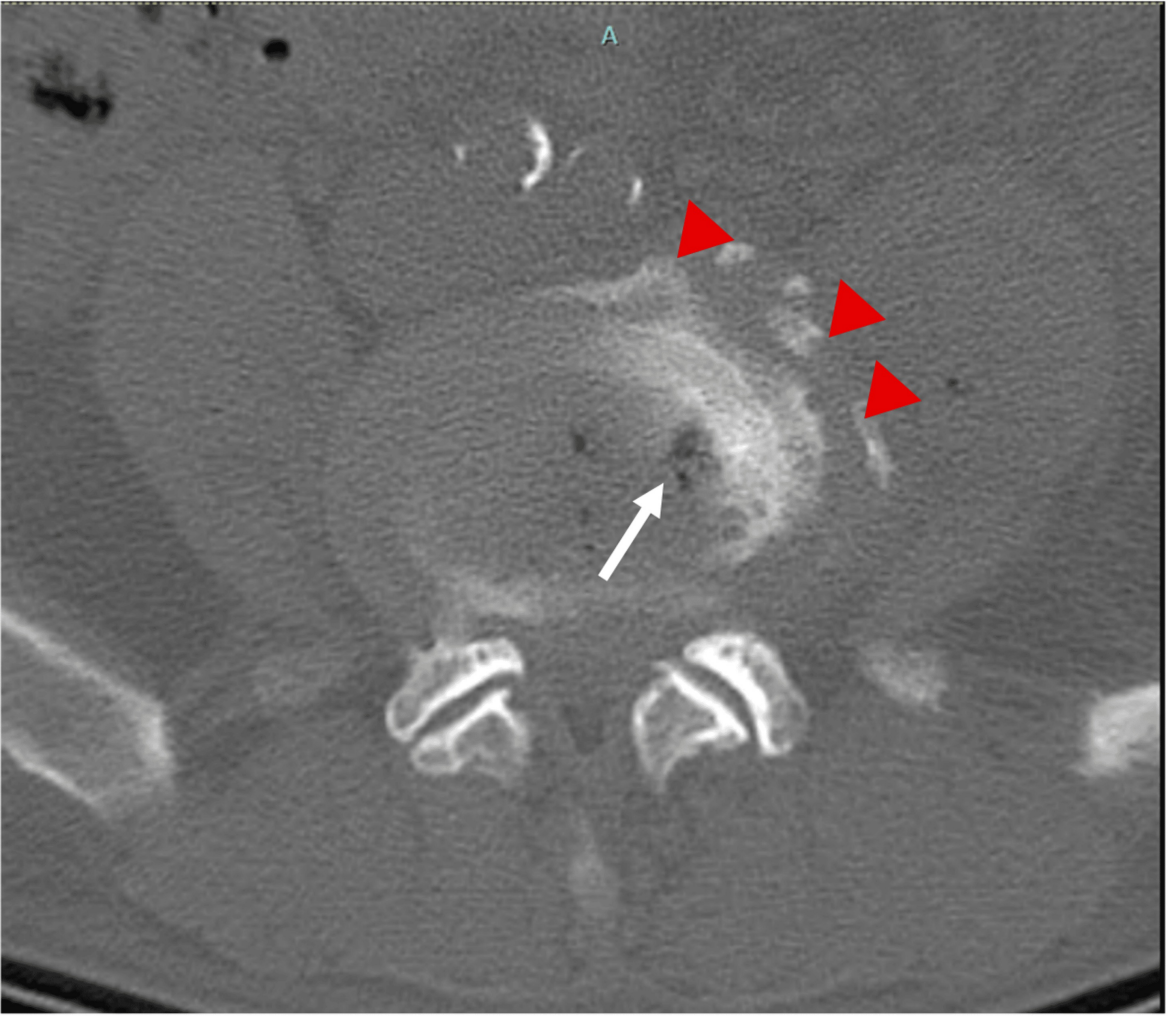
Non-contrast lumbar CT Axial view at the level of L4-L5 showing destruction and fragmentation of the vertebral body (red arrowheads), findings suggestive of an infectious process. Gas within the disk space may occasionally occur with infection (white arrow), although it is less common. CT - Computed tomography

A CT scan of the lumbar spine, despite not being the most sensitive or specific diagnostic test, revealed findings suggestive of spondylodiscitis (Figures 1, 2), prompting the initiation of empirical antibiotic therapy with ceftriaxone 2 g every 12 hours and vancomycin 750 mg every 12 hours [6]. Antibiotic therapy was later adjusted to ampicillin 3 g every six hours after *Streptococcus suis* was isolated from two blood cultures. However, after starting treatment, the patient developed a maculopapular rash, leading to discontinuation of the beta-lactam antibiotic and resumption of vancomycin therapy.

**Figure 3:**
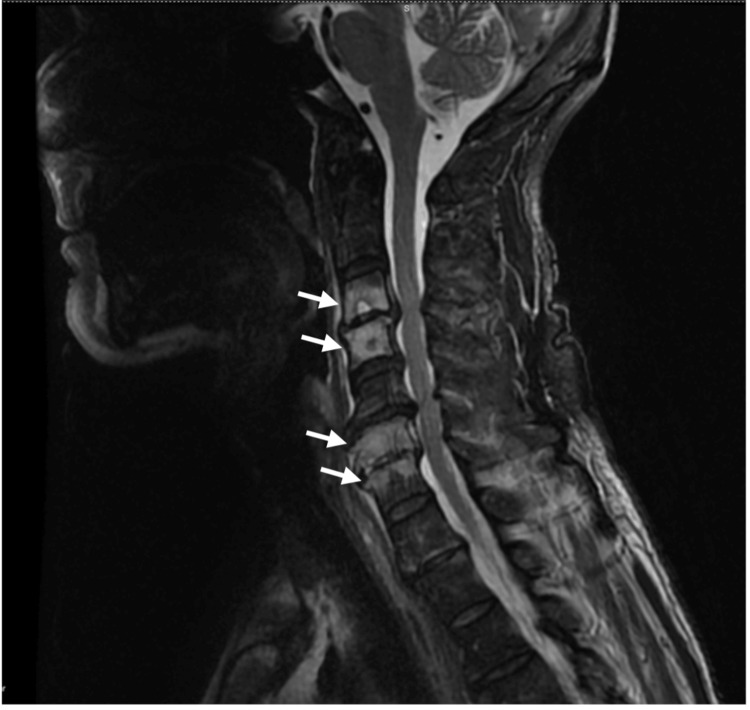
MRI, sagittal T2 of a 57-year-old male with infectious spondilodiscitis of C3, C4, C6, and C7 (white arrows) MRI - Magnetic resonance imaging

**Figure 4:**
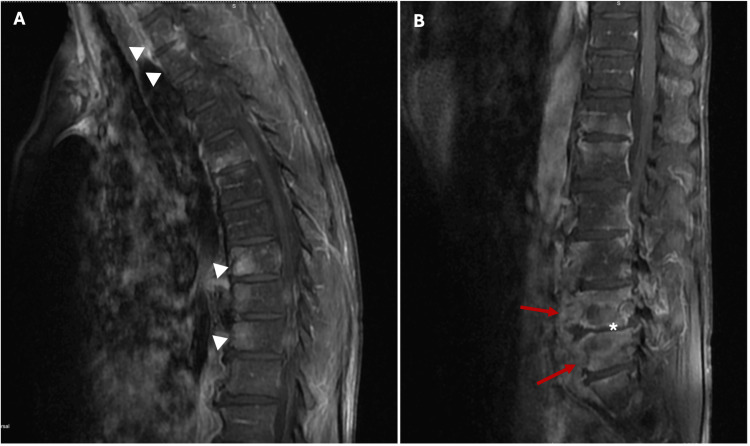
MRI of the dorsal and lumbar spine in a 57-year-old male with infectious spondylodiscitis A - Sagittal T1-weighted image of the dorsal spine; there is signal alteration with contrast enhancement after gadolinium injection in the D1, D2, D8, and D10 vertebrae (white arrowheads). B - Sagittal T1-weighted image of lumbar spine, L4 and L5, showing involvement of the somatic regions (red arrows), collapse of the disc space (asterisks), and a paravertebral tissue mass extending from L2 to S1. MRI - Magnetic resonance imaging

MRI revealed multisegmental spondylodiscitis involving the cervical, dorsal, and lumbar spine, with the greatest involvement of the dorsal and lumbar vertebral elements. No intracanal changes with compression of the cervical, dorsal, or conus medullaris were observed. There was mild meningo-radicular compression from L3 to L5 (Figures 3, 4).

Due to the lack of clinical and analytical improvement, a transthoracic and transesophageal echocardiogram was performed, which excluded endocarditis. Two CT-guided spinal biopsies were performed, both inconclusive, consistent with the literature reporting diagnostic rates of less than 40% for this technique [8].

After four weeks, due to difficulty maintaining therapeutic vancomycin levels and persistently elevated inflammatory markers, the therapy was switched to linezolid (600 mg every 12 hours). After completing six weeks of treatment, the patient showed clinical, analytical, and imaging improvement, including a reduction in ESR to 19 mm and a negative CRP.

The patient was followed up in the outpatient Internal medicine consult and underwent a follow-up MRI of the spine eight months after the initial MRI, showing favorable progression of the multifocal spondylodiscitis of the cervical and thoracolumbar spine, without signs of myelopathy.

## Discussion

Spondylodiscitis affects both the intervertebral disc and adjacent vertebral bodies [2]. It accounts for 3-5% of all cases of osteomyelitis, with a mortality rate that can reach 11% [9,10]. The infection can reach the spine by a) hematogenous dissemination (via the arterial or Batson venous plexus), b) direct external inoculation, and c) extension from adjacent affected tissues [11].

The incidence of sterile microbiological cultures ranges from 21% to 41.7% [12,13]. The most common etiological agents are *Staphylococcus aureus (*20-85%), coagulase-negative *Staphylococcus* (5-16%), *Streptococcus *and* Enterococcus *(5-20%), *Enterobacteriaceae* (7-33%), anaerobes (<4%), and polymicrobial infections (<10%) [14]. In the presented clinical case, spondylodiscitis occurred due to bacteremia from *S. suis*, related to the patient’s occupational activity, with embolization to the vertebral spine.

Regarding antibiotic therapy, the allergic reaction to the beta-lactam antibiotic limited the ability to use the most appropriate treatment. Therefore, it would be reasonable to consider performing antibiotic desensitization procedures to allow the use of a narrower-spectrum antibiotic with fewer potential complications.

CRP and ESR are reliable indicators for monitoring during the initial phase of the disease. As for imaging studies, MRI is the most sensitive diagnostic tool but is not recommended for monitoring treatment response. Bone scintigraphy and positron emission tomography (PET) are not routinely recommended for diagnosis or therapeutic monitoring [6].

Empirical antibiotic therapy typically consists of ceftriaxone 2 g IV every 12 hours in combination with vancomycin 15-20 mg/kg IV every 8-12 hours, adjusted based on serum levels (for bone infection and monitor renal function), until the etiological agent is identified. The duration of antibiotic therapy should be individualized but is generally maintained for a minimum of four to eight weeks [6]. In cases of neurological deficits, spinal instability, bone destruction, or abscess formation, evaluation by neurosurgery and/or orthopedics is essential, considering the need for surgical intervention [4].

## Conclusions

Spondylodiscitis is a serious and disabling condition with high mortality, despite advances in medical and surgical treatment. It should be considered in patients presenting with low back pain, radiculopathy, or myelopathy associated with fever and elevated inflammatory markers. Additionally, it should be considered in patients with comorbidities that predispose them to infections, such as diabetes, immunosuppressive conditions, chronic kidney disease, or those with a history of intravenous drug use or recent spinal procedures. Early diagnosis and empirical treatment are crucial for a good prognosis and recovery.
